# Mental Health Services Data Dashboards for Reporting to Australian Governments during COVID-19

**DOI:** 10.3390/ijerph181910514

**Published:** 2021-10-07

**Authors:** Sonam Shelly, Emily Lodge, Carly Heyman, Felicity Summers, Amy Young, Jennifer Brew, Matthew James

**Affiliations:** Australian Institute of Health and Welfare, Canberra 2617, Australia; sonam.shelly@aihw.gov.au (S.S.); emily.lodge@aihw.gov.au (E.L.); carly.heyman@aihw.gov.au (C.H.); amy.young@aihw.gov.au (A.Y.); jennifer.brew@aihw.gov.au (J.B.); matthew.james@aihw.gov.au (M.J.)

**Keywords:** mental, services, pandemic, COVID-19

## Abstract

The Australian Institute of Health and Welfare (AIHW) has been providing support to the Australian Government Department of Health to report on mental health-related data to Australian governments on a frequent basis since April 2020 in the form of COVID-19 mental health services data dashboards. These dashboards feature extensive use of data visualizations which illustrate the change in mental health service use over time as well as comparisons with pre-pandemic levels of service use. Data are included from the Medicare Benefits Schedule (MBS), Pharmaceutical Benefits Scheme (PBS/RPBS), Australian Government-funded crisis and support organizations, and key findings from emerging research. Demand for telehealth, crisis and support organizations and online mental health information services, in particular, have increased during the pandemic. The dashboards incorporate both new and existing data sources and represent an innovative way of reporting mental health services data to Australian governments. The reporting has enabled timely, targeted adjustments to mental health service delivery during the pandemic with improved cooperative data sharing arrangements having the potential to yield ongoing benefits.

## 1. Introduction

The global impact of the COVID-19 pandemic on mental health and wellbeing has been significant. The potential for this impact was recognized early in the pandemic and includes the direct health impacts of COVID-19 and fear of contagion, as well as the broader social and economic disruption [1].

In the Australian context, despite having experienced some significant COVID-19 outbreaks, containment measures have meant that the epidemic to date (July 2021) has generally been less severe than in many countries. However, the social, economic and mental health and wellbeing impacts of restrictions have been significant. Social distancing, sudden and protracted ‘lockdowns’, interruption of physical and mental health service provision, loss of employment, restricted international travel and border quarantine, and remote school and work have all had significant consequences. The impact of the pandemic in Australia started directly after an extreme drought and bushfire season for the country, a time of heightened anxiety for much of the population [2]. Data about the mental health effects of the pandemic at a national and international level are still evolving.

The Australian Institute of Health and Welfare (AIHW) has a long history in reporting mental health data, particularly through the Mental Health Services in Australia report and more recently the National Suicide and Self-harm Monitoring Project, a collaboration between the AIHW, the National Mental Health Commission and the Australian Government Department of Health (DoH), funded by the DoH. One of the key goals of this project is to improve the timeliness of state and territory data on suspected deaths by suicide.

A range of Australian research and reporting has been taking place on the mental health impacts of COVID-19 since early 2020. Some significant efforts include the Australian National University’s COVID-19 Impact Monitoring Survey Program, a longitudinal survey for which the AIHW provides financial support. This project seeks to monitor the economic and social wellbeing impacts of COVID-19 and is conducted through the ANUPoll, an ongoing quarterly probability-based panel survey of Australian public opinion. The availability of longitudinal pre-pandemic data from the ANUPoll facilitates analysis of the factors that have contributed to pandemic driven changes in psychological distress. The Australian Bureau of Statistics (ABS) has also been conducting surveys to investigate the impacts of the COVID-19 pandemic. From April 2020 to June 2021, the monthly Household Impacts of COVID-19 Survey collected longitudinal data on a range of topics including psychological distress. The Melbourne Institute (University of Melbourne) also commenced the Taking the Pulse of the Nation survey in April 2020.

Data were collected on a broad range of measures of mental health and wellbeing during the pandemic in Australia, including suicide and self-harm [3], life satisfaction, anxiety and worry [4], social connection, personal stressors, self-reported mental health [5] and use of mental health services [6]. One particularly relevant measure to the utilization of mental health services is that of psychological distress, commonly measured using the Kessler Psychological Distress Scale. A higher proportion of Australians have reported severe psychological distress during than pre-pandemic. Psychological distress and related measures tended to show peaks early in the pandemic and in conjunction with lockdowns, with elevated levels around April 2020 and again around August to October 2020 [4,5], the latter peak likely reflecting the impact of the relatively severe second wave in Victoria.

There was significant concern early in the pandemic about its potential impact on deaths by suicide [7], in part based on research linking unemployment to increased suicide rates [8]. Data on suspected deaths by suicide in 2020 for three Australian state suicide registers are included in the National Suicide and Self-harm Monitoring Project. This project seeks to improve the understanding of suicide and self-harm in Australia, to help identify factors that increase risk, to raise awareness and improve support and prevention activities. The AIHW routinely receives data for some states and territories, which is included in the dashboards. A key aim of the project is to establish registers for all Australian states and territories. To date, there has not been evidence of an increase in suspected deaths by suicide in any of these jurisdictions compared to previous years, however, the situation is complex and it remains important to continue to monitor the impact on suicide risk over time [3].

Analysis of ambulance attendances (one month per quarter data snapshots) available for some Australian states and territories from the National Ambulance Surveillance System (part of the National Suicide and Self-harm Monitoring Project) showed slight spikes in the population rate of ambulance attendances for self-injury and suicidal ideation during the outbreak period of the September 2020 quarter in Victoria. There was also a gradual increase in both measures between December 2019 and December 2020, particularly self-injury (4.7 to 6.5 per 100,000 population), although the rate of attendances for suicide attempts fell from 14.9 to 12.4 in the state over that year. Gradual increases in the rate of attendances for self-injury were also evident in the Australian Capital Territory (6.8 to 10.0) and Tasmania (2.1 to 5.9) over the period [9], however, whether these changes were related to the pandemic is unclear.

The impacts of the pandemic have not been evenly experienced across the Australian population. People with pre-existing mental health conditions [2] and few social supports [1] are at increased risk of distress. In general, younger Australians [10] and women have tended to have worse mental health outcomes than other Australians during the pandemic. Among young people (aged 44 and under), average psychological distress scores were elevated in 2020 compared to 2017, with the greatest increases for those aged 18–24 years. Women and people living in Victoria were the main drivers of an increase in psychological distress from May to August 2020 [11]. High mental distress (defined as ‘feeling depressed’ and/or ‘anxious’ ‘most or all of the time’) among parents also increased, from 8% in 2017 to 24% in 2020 [12].

There is evidence that the economic downturn associated with the pandemic has increased levels of psychological distress, with those employed in April 2020 having significantly lower levels of psychological distress than the unemployed at that time, when considering only those employed in February 2020 [13]. Rates of mental distress were approximately four times higher for people experiencing financial stress (42%) compared to people not experiencing financial distress (11.5%) during April to November 2020 [12].

The OECD has identified a similar range of increased risk factors associated with the pandemic which have contributed to a worsening of mental health, including unemployment and financial insecurity, reduced social connections, difficulties associated with telework, home schooling and education, restricted exercise and reduced access to health services. Peaks of mental distress have been closely related to waves of COVID-19 cases when restrictions have been most stringent [14]. The use of data visualizations in recent OECD reporting clearly illustrates some of the mental health impacts of COVID-19 across nations, particularly regarding depression and anxiety both during the pandemic and in comparison to pre-pandemic periods [14].

The mental health services system in Australia is complex and varies by state and territory, with some services funded by the Australian Government, others by state and territory governments or both. Mental health services are provided through public and private hospitals, residential and community mental health care, by specialist psychiatric and general medical practitioners, mental health nurses, psychologists and other allied health professionals. There are a range of crisis support services, as well as services provided through the National Disability Insurance Scheme and the non-government sector [15]. Australia’s federated model of health care also means that there is no single ‘master’ data set relating to health services.

Due to the complexity of the Australian mental health system and the need for timely data collection and analysis to guide the provision of mental health services during the pandemic [16], the AIHW has been providing support to the DoH to report on mental health-related data to Australian governments since April 2020, funded by the DoH. The COVID-19 National Mental Health Services dashboard and later the State and Territory Mental Health Services dashboard, were produced weekly during 2020, and fortnightly in 2021. These reports provide summary statistics of recent data and comparisons with the same data from early and pre-pandemic periods and extensive use of data visualizations of change over time. Data sources include information from the Medicare Benefits Schedule (MBS), Pharmaceutical Benefits Scheme (PBS) and Repatriation Pharmaceutical Benefits Scheme (RPBS), Australian Government-funded crisis and support organizations, and a brief summary of emerging research. A publicly available summary of the data is reported in the AIHW online publication, Mental Health Services in Australia [6], updated quarterly.

The aim of the present paper is to describe the background, development process, key results and learnings from the compilation of the data for the National Mental Health Services and State and Territory Mental Health Services dashboards. The processes that have led to success, and improvements in communication and data sharing within and across government and non-government organizations have broader implications for future health data, information and policy development.

## 2. Materials and Methods

### 2.1. Environment Scan

An environment scan and literature review were conducted to determine the range of Australian research and data holdings that were being established in relation to COVID-19 and mental health. During the initial scoping for the dashboard reporting, it was recognized that some of the organizations that would first see the impact of the pandemic on service utilization were those for which there was no national data collection, in particular, crisis and support organizations and online mental health information services.

### 2.2. Data Sources

#### 2.2.1. Crisis and Support Organizations and Online Mental Health Information Services

There are a number of Australian phone and online crisis and support services available to people seeking support for mental health issues. Crisis and support organization data include call, web chat, ‘app’ use, online programs and forums, and/or email data from a range of organizations, including Lifeline, Kids Helpline, Beyond Blue, Smiling Mind’s Healthcare Worker Program, Head to Health, Black Dog Institute and ReachOut. Data for HeadtoHelp hubs (Victorian Mental Health Clinics) also include face-to-face contacts.

#### 2.2.2. Medicare Benefits Schedule and Pharmaceutical Benefits Scheme Data

Services Australia collects fee-for-service related MBS claims activity data which it supplies to the DoH [17]. The Australian Government introduced additional MBS telehealth items during the COVID-19 pandemic, including items for mental health services provided by psychiatrists, GPs, allied health professionals and psychologists. MBS subsidized services under the Better Access to Psychiatrists, Psychologists and General Practitioners through the MBS (Better Access) initiative were also expanded [18]. MBS data reported in the dashboards include use of MBS mental health items (services processed), the proportion of services delivered via telehealth, and MBS benefits paid.

The Australian Government subsidizes the cost of prescription medicines through two schemes, the PBS and RPBS for eligible veterans and their dependents. Services Australia processes all prescriptions dispensed under the PBS/RPBS and provides these data to the DoH [19]. PBS/RPBS data reported includes the number of PBS dispensed mental health-related prescriptions.

Further information on these data sources is available at https://www.aihw.gov.au/reports/mental-health-services/mental-health-services-in-australia/ (accessed on 5 October 2021).

#### 2.2.3. Emerging Research

Key points from emerging research are provided in each dashboard update, with a more detailed discussion in the Mental Health Services in Australia quarterly online update. This includes key findings from research programs outlined in the introduction to this article.

### 2.3. Data Access and Analysis

#### 2.3.1. Data Access and Metric Selection

The data supply from crisis and support organizations and online mental health information services to the AIHW was established in collaboration with the DoH, facilitated through their existing contractual arrangements with the agencies. A prototype data collection template was prepared by the AIHW, with adjustments made as required for individual agency collections. The collection includes daily data on contact volumes and weekly aggregate data on some demographic variables including age, sex, Indigenous status, state or territory and reason for call. Due to data quality issues, reporting of demographic variables (particularly Indigenous status and age) has been limited.

Under an existing arrangement with the DoH, the AIHW had access to the MBS and PBS/RPBS data via the Department’s Enterprise Data Warehouse (EDW) for use in AIHW’s regular reporting products. A list of MBS item numbers relating to mental health was identified, based largely on the AIHW’s existing Mental health services in Australia reporting. Early analysis identified key metrics for reporting, which have been refined over time. Mental health-related prescriptions in the PBS/RPBS data were identified using Anatomic Therapeutic Chemical (ATC) codes and ongoing trend analysis included from November 2020.

The dashboard was initially funded and prepared for a national view of mental health impacts. Clear gaps became evident early in the process, significantly that the dashboard only included data from Australian Government funded services; it was missing data from the public mental health system run by state and territory governments. In addition, situations in each state and territory have varied markedly, with the majority of cases occurring in two states, Victoria and New South Wales. On behalf of the DoH, the AIHW established processes to share data across levels of government and developed an agreement to include data at the state and territory level. Data sharing initially focused on the two most populous states of New South Wales and Victoria (together comprising 58% of the total population) which had the largest COVID-19 case numbers (87% of all cases to April 2021) [20]. Agreements were established by the AIHW on behalf of the DoH, for participating states to provide emergency department, community mental health care services and admitted patient (hospitals) data at the required intervals, with the AIHW providing MBS and crisis and support organization data back to these states for their own reporting and monitoring purposes. Queensland subsequently joined the data sharing arrangement and was included in the State and Territory Mental Health Services dashboard from May 2021. Data for Queensland will be included in AIHW’s reporting on Mental health service in Australia from October 2021.

#### 2.3.2. Data Analysis

Data were analysed in SAS Enterprise Guide 7.1 and Microsoft Excel^©^. Data visualizations were created in Tableau software version 2020.3. The data were presented as A3 colour posters, with data and graphics grouped by data source.

There are hundreds of mental health-related MBS items, and items are often added and removed from the schedule. The list of items analysed were similar to that described in Mental health services in Australia [21]. MBS data were analyzed by date of processing, as this results in more stable historical values over time. During code development, the extraction method was validated by reviewing results against those obtained through the Australian Government Services Australia Medicare Item Reports tool [22]. When extracting data, it was also ensured that data existed up to the last date of interest, to ensure that analyses were not impacted by unanticipated delays to data warehouse updates.

PBS items analyzed included antipsychotics (N05A), anxiolytics (N05B), hypnotics and sedatives (N05C), antidepressants (N06A), and psychostimulants, agents used for ADHD and nootropics (N06B), according to the Anatomical Therapeutic Chemical (ATC) Classification System [23]. PBS data were lagged by at least 6 weeks from the extraction date to reduce the effect of late claims, updates and cancellations.

Statistics supplied by non-government organizations were routinely checked for consistency with historical supplies. Any changes to historical values were queried with the supplying organization, and then corrected if necessary. Time series were also routinely inspected manually for any anomalies or unusual patterns, which were also queried. Furthermore, these organizations may run their own data cleansing procedures from time to time, resulting in minor changes to historical values.

## 3. Results

The following is a summary of the publicly available results for data to 25 April 2021 for the COVID-19 National Mental Health Services Dashboard and limited data from the state level dashboard, published in Mental Health Services in Australia in July 2021 [6]. In addition to data visualizations and descriptions of trends over time, the following descriptions include comparisons of the most recent publicly available (at time of writing) month of data compared with the same month in 2020 and 2019. These comparisons are provided in the dashboards to help illustrate differences between pre-pandemic, early pandemic and more recent data.

### 3.1. Use of Medicare-Subsidised Mental Health-Related Services

**Figure 1:**
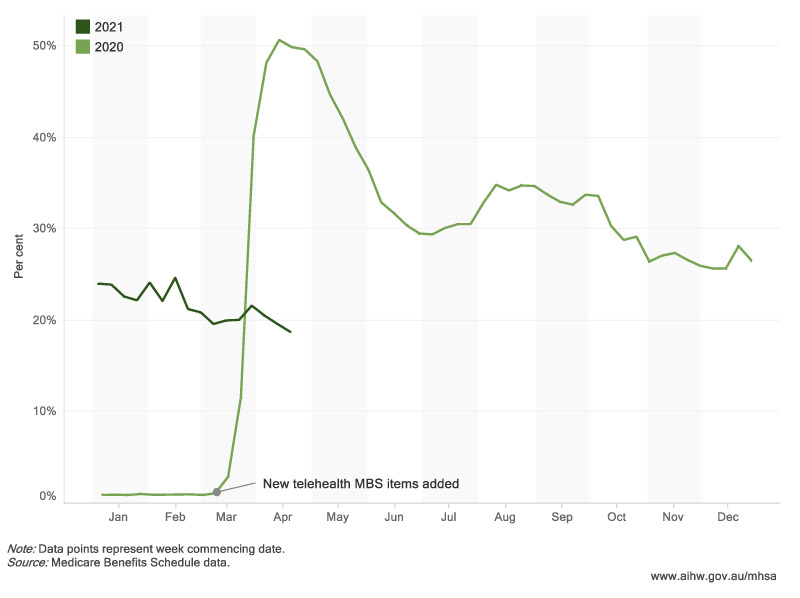
MBS mental health telehealth services (proportion of all MBS mental health services), January 2020–April 2021. (Website: https://www.aihw.gov.au/reports/mental-health-services/mental-health-services-in-australia/report-contents/covid-19-impact-on-mental-health, accessed on 5 October 2021).

- MBS mental health service usage showed a generally upward trend from early April 2020 to end of April 2021, with temporary dips observed during major holiday periods. Over 15.0 million MBS-subsidized mental health-related services were delivered between 16 March 2020 and 25 April 2021, with just under one third (29.5%) delivered by telehealth. The number of services delivered in the 4 weeks to 25 April 2021, was almost one fifth higher than the number of services provided in the same 4-week period in 2020 and 2019.
- Delivery of MBS subsidized mental health services via telehealth peaked in April 2020, when about half of these services were delivered remotely, corresponding with Australia’s national lockdown in April—May 2020 (Figure 1).

### 3.2. Pharmaceutical Benefits Scheme (PBS) Prescriptions

**Figure 2:**
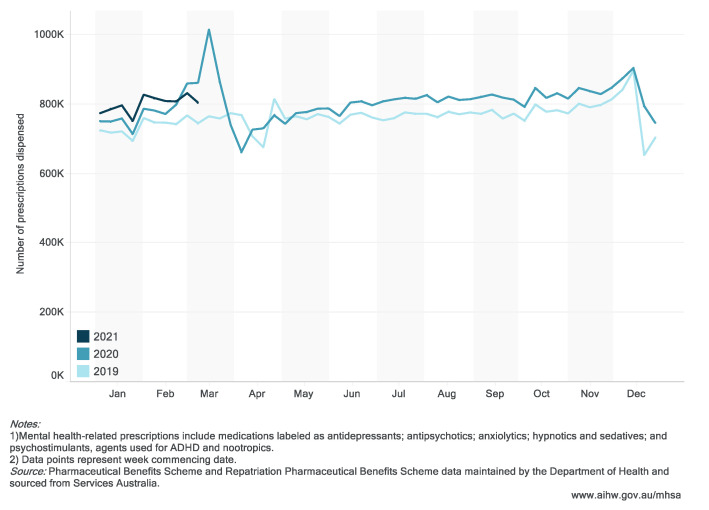
PBS mental health-related prescriptions dispensed (by week), January 2019–March 2021. (Website: https://www.aihw.gov.au/reports/mental-health-services/mental-health-services-in-australia/report-contents/covid-19-impact-on-mental-health, accessed on 5 October 2021).

- There was a spike in all mental health-related PBS-subsidized and under co-payment prescriptions in the 4 weeks to 29 March 2020 at the peak of Australia’s initial outbreak, an 18.6% increase in the number of prescriptions dispensed compared to the same period in 2019 (Figure 2).

### 3.3. Use of Crisis and Support Organisations and Online Mental Health Information Services

Crisis and support organizations and online mental health information services have reported significant demand increases during the pandemic. Calls to Lifeline increased in 2020 compared to 2019 and have stayed at an elevated level.

**Figure 3:**
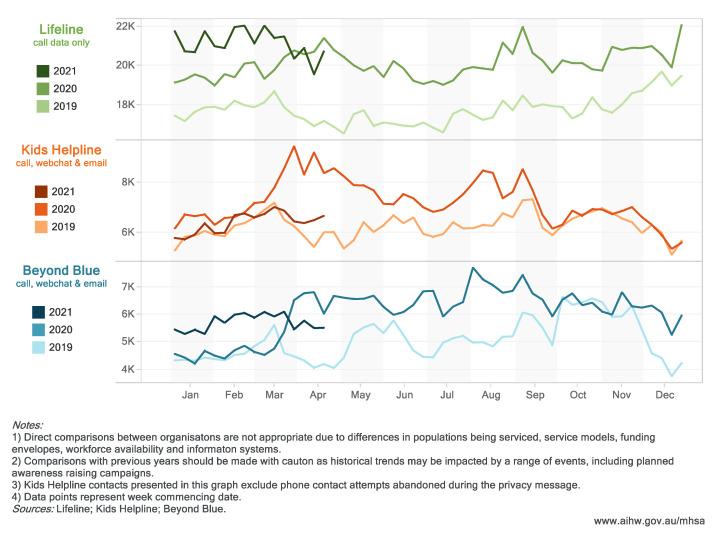
Crisis and support organization contacts (by week), January 2019–April 2021. (Website: https://www.aihw.gov.au/reports/mental-health-services/mental-health-services-in-australia/report-contents/covid-19-impact-on-mental-health, accessed on 5 October 2021).

Contacts with Beyond Blue increased in March 2020 and stayed elevated throughout the year, settling into a level between 2019 and 2020 volumes in March—April 2021. Kids Helpline contacts spiked in early April 2020, and trended down over the course of 2020, settling back to 2019 levels in 2021 (Figure 3).

In the 4 weeks to 25 April 2021 (compared to the same period in 2020 and 2019):

- Almost 82,000 calls were made to Lifeline, a similar volume (2.3% decrease) to April 2020 and an 18.4% increase from April 2019;
- Over 22,000 contacts were made with Beyond Blue, a 14.9% decrease and 30.7% increase from April 2020 and 2019, respectively;
- Approximately 26,000 contact attempts were made to Kids Helpline (not including those abandoned during the privacy message), a 26.6% decrease and 10.5% increase from April 2020 and 2019, respectively;
- Head to Health and ReachOut websites had an increase in visits at the start of the pandemic, with a peak in March 2020. ReachOut reported approximately 7600 daily website visits in April 2021, decreases of 34.5% and 13.2% compared to April 2020 and 2019, respectively. Head to Health reported about 1400 users per day in April 2021, a 74.7% decrease and 37.0% increase compared to the same periods in 2020 and 2019, respectively.

### 3.4. Jurisdictional Differences in Mental Health Service Activity

Data by some Australian states and territories is included in a state level dashboard, which is supplementary to the national dashboard. Publicly available reporting on this jurisdictional dashboard is currently only available for New South Wales and Victoria.

Australian states and territories have experienced varying levels of outbreaks over the course of the pandemic, striking at different time periods, ranging from no community transmission to the large ‘second wave’ outbreak in Victoria in winter 2020. Pandemic outbreaks and associated restrictions show clear patterns in the use of MBS telehealth services and use of crisis and support organizations.

**Figure 4:**
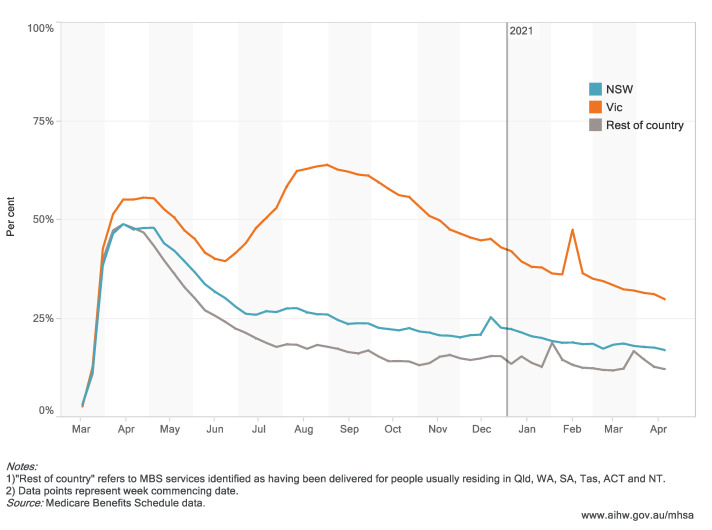
MBS mental health telehealth services (proportion of all MBS mental health services), by jurisdiction, March 2020–April 2021. (Website: https://www.aihw.gov.au/reports/mental-health-services/mental-health-services-in-australia/report-contents/covid-19-impact-on-mental-health, accessed on 5 October 2021).

- The population rate of MBS mental health services was generally higher throughout the course of 2020 than during 2019, in both states, with the differential most evident in Victoria from the second half of 2020 onwards. This elevated rate is still evident in 2021. This pattern was likely influenced by the introduction of 10 additional subsidized psychology sessions under the Better Access initiative to people living under lockdown initially, which was then expanded to all Australians.
- The 4 weeks to 13 September 2020 in Victoria and the 4 weeks to 7 March 2020 in New South Wales were the periods with the highest number of MBS mental health-related services with about 360,000 services in each state.
- In New South Wales, there was an initial steep increase in telehealth services between March and April 2020, followed by a gradual decline, consistent with the pattern for the country overall. Victoria showed a double peak in telehealth service use, consistent with the second wave in winter 2020, and a small but sharp spike in February 2021 at the time of a smaller outbreak in Victoria (Figure 4).
- There have been some clear jurisdictional differences since the beginning of the pandemic in the rate of crisis and support organization contacts. In Victoria, calls to Beyond Blue showed a notable spike, commencing in July 2020, consistent with the onset of their second wave. The rate of call volumes to Lifeline, Kids Helpline and Beyond Blue all showed a greater difference between 2019 and 2020 in Victoria than in New South Wales. However, Lifeline and Kids Helpline both showed a notable ongoing higher level of call volume in NSW in 2020 than in 2019. Lifeline calls in NSW in the early part of 2021 were notably higher, possibly related to the northern beaches outbreak at that time.

## 4. Discussion

The COVID-19 mental health services dashboards represent an innovative way of reporting high level mental health services data to Australian governments.

Much of the data included in the dashboards had not been reported in this way previously, including data from crisis and support organizations and online mental health information services. The dashboards include a variety of data in one view, over time, at frequent reporting intervals, giving a unique overview and triangulation of data sources that lead to additional insights and confirmation of patterns in service demand and use. Interactions and common movements across different types of service use are readily visible, demonstrating clearly the impacts of the pandemic relative to baseline results, and the influence of lockdowns, other restrictions and outbreaks evident in service use over time. This has enabled the data to form an integral part of the evidence base drawn on by Australian governments to determine adjustments to mental health service provision during the pandemic.

The frequent reporting has supported ongoing, timely reporting improvement both through internal review and frequent feedback from external stakeholders. High frequency reporting has been far more informative than annual reporting could be in the current context.

Ongoing communication with and between data receivers, providers and the analysis team has been crucial when unexpected changes in the data have been observed, so as to avoid spurious conclusions. In some cases, changes in service use have been due to planned service delivery changes rather than pandemic related demand changes.

Maintaining agility and being alert to shifting trends in the data has been an ongoing challenge, particularly given the tight timeframes inherent in weekly and fortnightly reporting. Consideration is being given to the potential benefits of presenting the dashboard material as a restricted release online webpage in future, to allow greater interaction for end users and more flexibility in presentation of content.

As noted above, the present description of the dashboard results is limited to those publicly available at the time of writing. Ongoing updates to the data are published on a quarterly basis in the Mental health services in Australia online publication.

It should be noted that while reporting of aggregate data on mental health service use provides useful insights into the utilization of mental health services, it cannot provide information on either the adequacy of services or the benefits of treatment at the individual level. Neither should it be assumed that the pandemic is the underlying reason for all changes in mental health service use over the reporting period.

There is no ‘master’ data set for reporting on the use of mental health services in Australia. The data presented in the dashboards are limited to aspects of the mental health system for which adequate data are available or could be readily developed for reporting on a frequent (i.e., at least fortnightly) basis. The AIHW will continue to work to improve available data on the use of mental health services.

## 5. Conclusions

The reporting of data on mental health service use in Australia is evolving. While large administrative data sets are a valuable source of epidemiological data, traditionally long lead times have often limited their utility for governments responding to time sensitive issues. The use of administrative data to inform policy responses to emerging challenges, notably, disaster recovery and the impact of the COVID-19 pandemic, has required governments to adapt existing protocols, particularly relating to data timeframes, to meet these new needs.

The COVID-19 mental health services dashboard project and associated reporting has involved new levels of data sharing and communication between Australian and state and territory governments, and non-government organizations on the utilization of mental health services in Australia. The project has demonstrated willingness and ability of these organizations to implement cooperative data sharing arrangements that have the potential to lead to ongoing benefits that extend beyond the pandemic.

## Data Availability

The de-identified unit record datasets used to generate the present analyses are not publicly available. Further data related to the mental health COVID-19 dashboards is available at https://www.aihw.gov.au/reports/mental-health-services/mental-health-services-in-australia/report-contents/mental-health-impact-of-COVID-19 (accessed on 5 October 2021).
